# Supplementary material for: Cinnamaldehyde-Treated Bone Marrow Mesenchymal-Stem-Cell-Derived Exosomes via Aqueous Two-Phase System Attenuate IL-1β-Induced Inflammation and Catabolism via Modulation of Proinflammatory Signaling Pathways
Source: Int J Mol Sci. 2024 Jul 1;25(13):7263. doi: 10.3390/ijms25137263 (PMC11242605; doi:10.3390/ijms25137263)
Supplement: Supplementary file 1 [file ijms-25-07263-s001.zip › ijms-3033616-supplementary.pdf]

Cinnamaldehyde (CA) Treatment to Chondrocytes

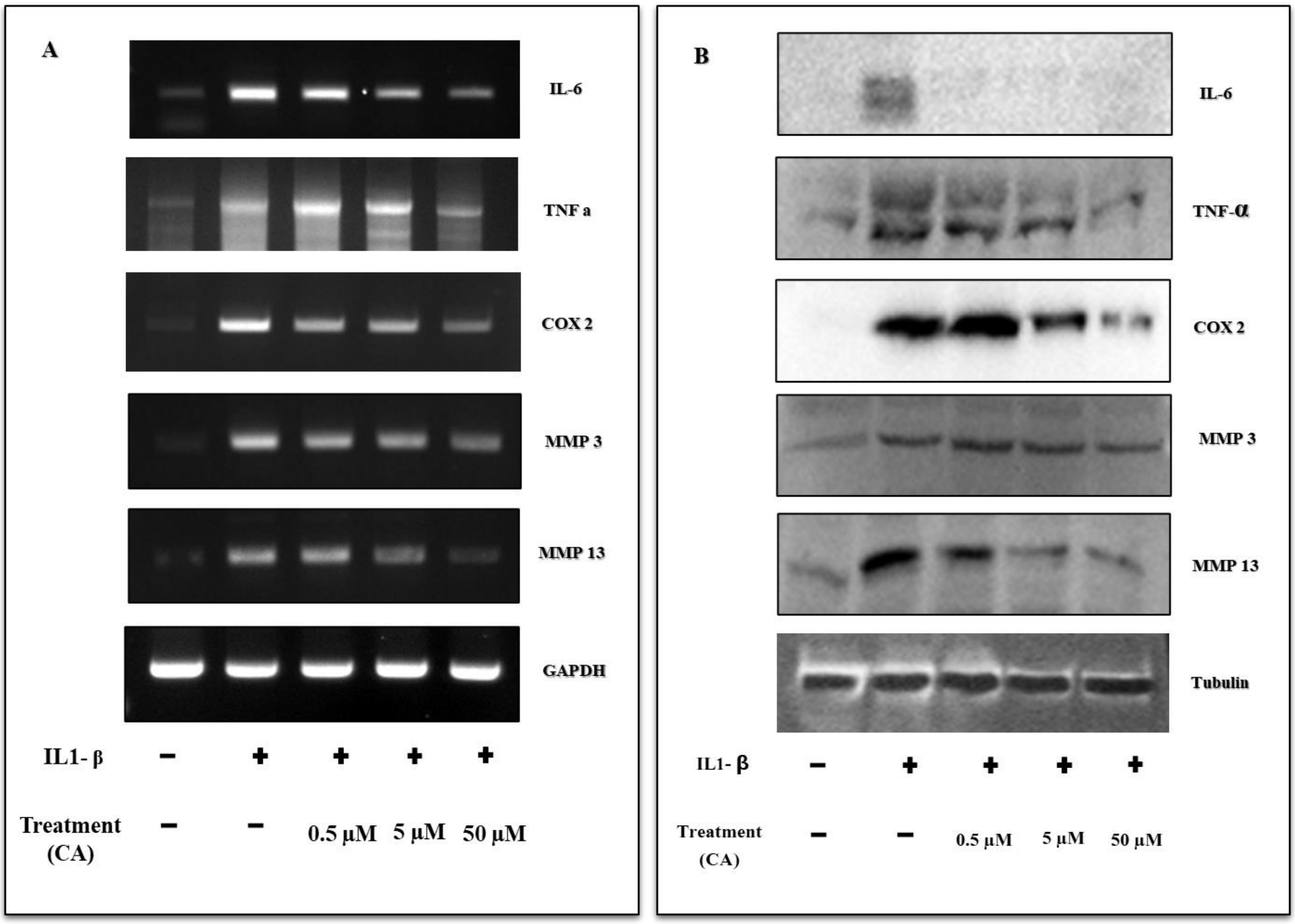

Supplementary Figure S1. Effects of Cinnamaldehyde on inflammation specific mediators in a dose-dependent manner. (A) Gene

expression levels of IL-6, TNF-α, COX 2, MMP 3, and MMP 13 were analyzed using RT-PCR (B) Protein expression levels of

IL-6, TNF-α, COX 2, MMP 3, and MMP 13 were analyzed using Western blot.

Cinnamaldehyde treatment to cells to determine the cellular toxicity.

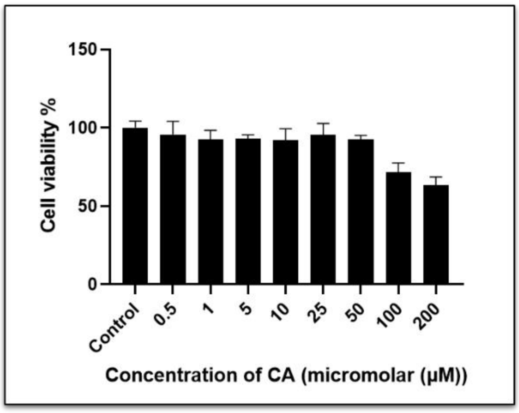

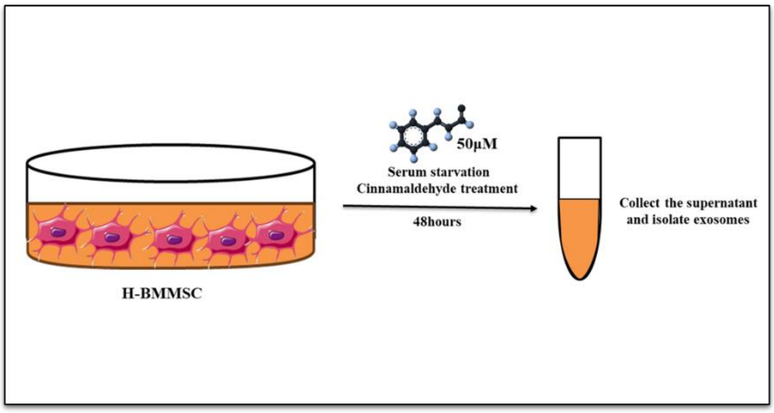

Supplementary Figure S2. Cellular cytotoxicity of chondrocytes treated with different concentrations of cinnamaldehyde (CA). The optimum concentration of cinnamaldehyde (CA) for treatment of chondrocytes appears to be 50 μM, as it does not significantly impact cell viability while still providing potential therapeutic effects.

Live-Dead staining assay

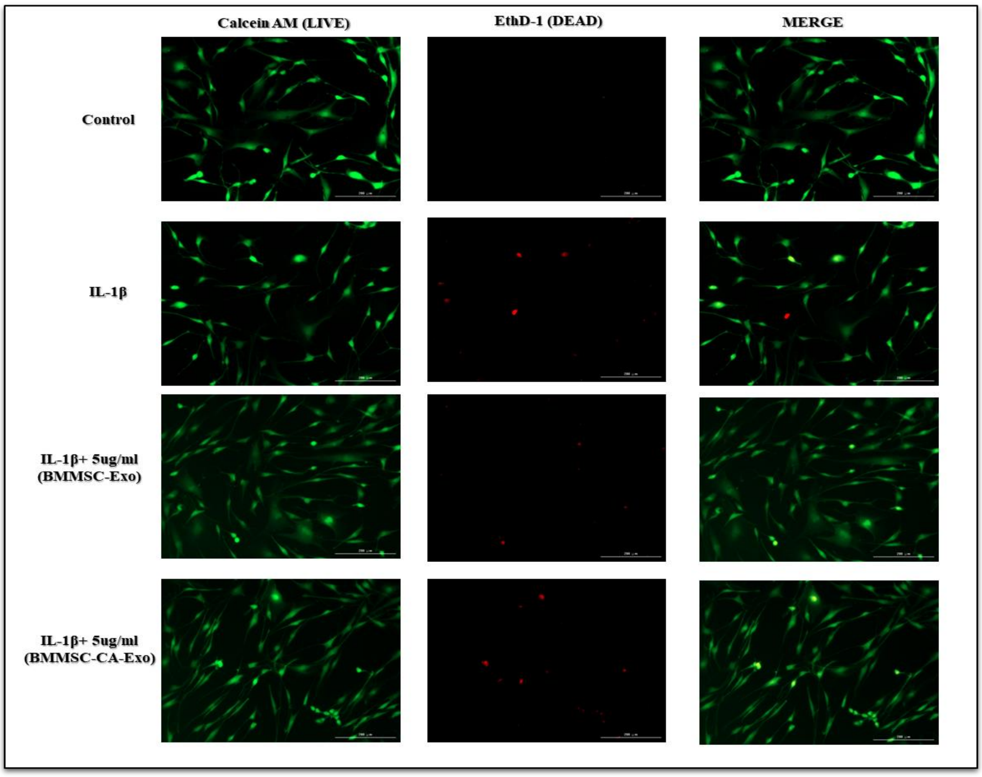

Supplementary Figure S3. Live-dead staining assay of chondrocytes using calcein and EthD-1 under different treatment conditions. The control group with no treatment shows no dead cells. The inflammation group stimulated with IL-1β has significantly more dead cells stained by EthD-1. Treatment with BMMSC-derived exosomes (BMMSC-Exo) decreases the number of dead cells compared to the IL-1β group. Treatment with cinnamaldehyde-activated BMMSC-derived exosomes (BMMSC-CA-Exo) also reduces the number of dead cells compared to the IL-1β group, to a greater extent than BMMSC-Exo alone. These results indicate that exosomes derived from bone marrow mesenchymal stem cells, particularly those pre-treated with cinnamaldehyde, have a protective effect against inflammation-induced chondrocyte death.

Gene Expression (Primer details)

Primer Name	Forward primer	Reverse Primer	Melting temp
hCollagen II	TCTGCAACATGGAGACTGGC	GAAGCAGACCGGCCCTATGT	F 55.7<br>R 57.6
hIL6	GGATGCTTCCAATCTGGATTCAA<br>TGAG	CGCAGAATGAGATGAGTTGTCAT<br>GTCC<br>\	F 63.5<br>R 63.6
hCOX2	TTC AAATGAGATTGTGGGAAA	AGATCATCTCTGCCTGAGTATCTT	F 51.9<br>R 52.0
hMMP13	GATGAAGACCCCAACCCTAAA	CTGGCCAAAATGATTTCGTTA	F 54.3<br>R 54.1
hGAPDH	ACCACAGTCCATGCCATCAC	TCCACCACCCTGTTGCTGTA	F 55<br>R 55.9

Supplementary Table S1. The primer sequences used in the following research.
